# Diagnostic performance of the (1–3)-β-D-glucan assay in patients with *Pneumocystis jirovecii* compared with those with candidiasis, aspergillosis, mucormycosis, and tuberculosis, and healthy volunteers

**DOI:** 10.1371/journal.pone.0188860

**Published:** 2017-11-30

**Authors:** Hyo-Ju Son, Heungsup Sung, Se Yoon Park, Taeeun Kim, Hyun Jeong Lee, Sun-Mi Kim, Yong Pil Chong, Sang-Oh Lee, Sang-Ho Choi, Yang Soo Kim, Jun Hee Woo, Sung-Han Kim

**Affiliations:** 1 Departments of Infectious Diseases, Asan Medical Center, University of Ulsan College of Medicine, Seoul, Republic of Korea; 2 Laboratory Medicine, Asan Medical Center, University of Ulsan College of Medicine, Seoul, Republic of Korea; Food and Drug Administration, UNITED STATES

## Abstract

**Background:**

Diagnosis of pneumocystis pneumonia (PCP) relies on microscopic visualization of *P*. *jirovecii*, or detection of Pneumocystis DNA in respiratory specimens, which involves invasive procedures such as bronchoalveolar lavage. The (1–3)-β-D-glucan (BG) assay has been proposed as a less invasive and less expensive diagnostic test to rule out PCP. We therefore compared blood levels of BG in patients with PCP with those of patients with candidemia, chronic disseminated candidiasis (CDC), invasive aspergillosis, mucormycosis, and tuberculosis and those of healthy volunteers.

**Methods:**

Adult patients who were diagnosed with PCP, candidemia, CDC, invasive aspergillosis, mucormycosis, and tuberculosis whose blood samples were available, and healthy volunteers were enrolled in a tertiary hospital in Seoul, South Korea, during a 21-month period. The blood samples were assayed with the Goldstream Fungus (1–3)-β-D-glucan test (Gold Mountain River Tech Development, Beijing, China).

**Results:**

A total of 136 individuals including 50 patients *P*. *jirovecii*,15 candidemia, 6 CDC, 15 invasive aspergillosis, 10 mucormycosis, and 40 controls (20 TB and 20 healthy volunteers) were included. The mean±SD of the concentration of 1–3-β-D-glucan in the patients with PCP (290.08 pg/mL±199.98) were similar to those of patients with candidemia (314.14 pg/mL±205.60, *p* = 0.90 at an α = 0.005) and CDC (129.74 pg/mL±182.79, *p* = 0.03 at an α = 0.005), but higher than those of patients with invasive aspergillosis (131.62 pg/mL±161.67, *p* = 0.002 at an α = 0.005), mucormycosis (95.08 pg/mL±146.80, *p*<0.001 at an α = 0.005), and tuberculosis (103.31 pg/mL±140.81, *p*<0.001 at an α = 0.005) as well as healthy volunteers (101.18 pg/mL±197.52, *p*<0.001 at an α = 0.005). At a cut-off value > 31.25 pg/mL, which is highly sensitive for PCP versus tuberculosis plus healthy volunteers at the expense of specificity, the BG assay had a sensitivity of 92% (95% CI 81%-98%) and a specificity of 55% (95% CI 39%-71%).

**Conclusions:**

The BG assay appears to be a useful adjunct test for PCP.

## Introduction

Pneumocystis pneumonia (PCP) is one of the most frequent opportunistic infections in immunocompromised patients [1]. The mortality of patients requiring mechanical ventilation ranges from 50%to 60% [2]. This high mortality rate results partly from difficulties in early diagnosis because of the nonspecific clinical features, concurrent use of prophylactic drugs, and possible coinfections with other microorganisms [1, 3]. Because *P*. *jirovecii* cannot be propagated in culture, the standard procedures for detecting this microorganism are microscopic visualization of cysts or trophic forms in pulmonary specimens along with cytochemical or immunofluorescence staining with monoclonal antibodies, or DNA amplification (PCR or PCR-related protocols) [4]. However, acquiring pulmonary specimens usually involves invasive techniques such as bronchoaveolar lavage (BAL), which can be difficult especially in critically-ill patients. Therefore, rapid and non-invasive diagnostic tests are needed [5–8].

In addition, the incidence of invasive fungal infections (IFIs) such as candidiasis, aspergillosis, and mucormycosis has been steadily increasing as a consequence of factors such as aggressive cancer chemotherapy, bone marrow and organ transplantation, AIDS, and advances in critical care [9]. Early identification of IFIs is challenging because its accompanying signs and symptoms are nonspecific. Furthermore, fungal culture is not sensitive enough to rule out IFIs, and it also requires days to weeks of incubation, which may greatly delay initiation of antifungal therapy [10]. The determination of (1 → 3)-β-d-glucan (BG) serum levels is a non-invasive test for circulating fungal cell wall components that allows systematic screening and prompt identification of *P*. *jirovecii* and other fungal infections with the exception of cryptococcosis and mucormycosis [11, 12]. The BG assay has been proposed as a less invasive and inexpensive screening test for PCP and IFIs. We therefore compared the blood levels of BG in patients with PCP with those of patients with candidemia, chronic disseminated candidiasis (CDC), invasive aspergillosis, mucormycosis, and tuberculosis (TB), and of healthy volunteers.

## Materials and methods

### Study population

Adult patients diagnosed with PCP, invasive aspergillosis, mucormycosis, and CDC were enrolled in a tertiary hospital in Seoul, South Korea, between December 2014 and August 2016. In addition, blood samples were obtained from randomly selected patients with candidemia and TB, whose blood samples were available, and from healthy volunteers. The plasma was separated from each of the blood samples obtained from these patients or volunteers and was stored in a −80°C freezer. The study protocol was implemented in accordance with the principles of the Helsinki Declaration. Approval was obtained from the institutional ethics committee, and each patient provided written informed consent before participating in the study. The study was approved by the Institutional Review Board of the Asan Medical Center (IRB No. 2014–0198).

### Definitions

PCP was diagnosed based on a positive test result in an immunohistochemical (IHC) antibody assay (Dako, Santa Barbara, CA, USA) for *P*. *jirovecii* using BAL fluid in patients who had respiratory symptoms and radiological findings compatible with PCP. All the IHC tests for PCP were read by one experienced clinical microbiologist (H.S.). Proven aspergillosis was defined by histological evidence of tissue invasion consisting of septate, acutely branching filamentous fungi plus the recovery of *Aspergillus* species by the culture of pulmonary tissue, or positivity for IHC staining with an anti-*Aspergillus* monoclonal antibody (LSBio, Seattle, WA, US). Probable aspergillosis was defined by the presence of host factors together with one or more clinical indications such as dense, well-circumscribed lesions with or without a halo sign; an air-crescent sign or cavity on computed tomography (CT); and mycological evidence of fungal infection (by culture or cytological analysis of non-sterile material such as BAL fluid for *Aspergillus* species, or galactomannan assay of serum or BAL fluid). The above definition corresponds with the revised criteria of the European Organization for Research and Treatment of Cancer/Mycosis Study Group [13, 14]. Proven mucormycosis was defined by histological evidence of tissue invasion consisting of non-septate, right-angle-branching filamentous fungi, in addition to the recovery of Mucorales species by the culture of specimens from sterile material, or positivity for immunohistochemical staining with an anti-*Rhizopus arrhizus* monoclonal antibody (LSBio). Probable mucormycosis was defined by the presence of host factors together with one or more clinical indications, such as dense, well-circumscribed lesions with or without a halo sign; an air-crescent sign or cavity on CT; and mycological evidence of Zygomycetes in non-sterile material such as sputum or BAL fluid culture. The above definition corresponds to the revised criteria of the European Organization for Research and Treatment of Cancer/Mycosis Study Group [13, 14]. Candidemia was defined by the presence of at least one positive blood culture for *Candida* species in a patient with clinical signs and symptoms of sepsis. Proven CDC was defined by the finding of a yeast infection via the staining of liver biopsy samples according to standard laboratory methods or positive culture results from liver biopsy specimens and the liver and/or spleen exhibiting clinical/biological or radiologic symptoms consistent with infection. Probable CDC was defined by the patients having hematologic malignancies yielding small, peripheral, “target-like” CT images in the liver and/or spleen, and having elevated serum alkaline phosphatase levels without any evidence of an alternative diagnosis [15].

### The (1–3)-β-D-glucan test

BG is a polysaccharide present in the cell wall of most fungi including *Aspergillus*, *Candida*, *Fusarium Acremonium*, *Penicillium*, *Paecilomyces*, and *P*. *jirovecii*, but not bacteria or viruses [11]. The BG assay relies on the ability of BG to activate a coagulation cascade within amebocytes from the hemolymph of horseshoe crabs [16]. Blood samples were assayed with the Goldstream Fungus (1–3)-β-D-glucan test (GKT-12M; Gold Mountain River Tech Development). Values below the minimum detectable level (31.25 pg/mL) were recorded as 31.25 pg/mL, and those above the maximum detectable level (500 pg/mL) were recorded as 500 pg/mL.

### Statistical analysis

Statistical analyses were performed with SPSS for Windows (version 1221.0; SPSS Inc, Chicago, IL, USA). Diagnostic performance was expressed in terms of sensitivity, specificity, positive predictive value, negative predictive value, positive likelihood ratio, and negative likelihood ratio. Continuous variables were compared using the Kruskal–Wallis test. Pairwise comparisons were performed using the Mann–Whitney U test and the alpha level was adjusted to 0.005 under the Bonferroni criteria. All tests of significance were 2-tailed. The diagnostic accuracy of the BG assay was assessed by analysis of receiver operating characteristic (ROC) curves. The Youden index was used to select the optimum cut-off points on the ROC curves (optimal balance between sensitivity and specificity).

## Results

### Patient characteristics

A total of 136 individuals comprising 50 patients with *P*. *jirovecii* (47 non-HIV and 3 HIV), 15 with candidemia, 6 with CDC, 15 with invasive aspergillosis, 10 with mucormycosis, and 40 controls (20 patients with TB and 20 healthy volunteers) were included in the analysis. Of the 15 patients with invasive aspergillosis, 6 (40%) were classified as proven invasive aspergillosis and 9 (60%) as probable invasive aspergillosis. Of the 10 patients with mucormycosis, 8 (80%) were classified as proven invasive mucormycosis and 2 (20%) as probable invasive mucormycosis. Of the 20 patients with TB,9 (45%) had disseminated TB, 5 (25%) TB lymphadenopathy, 3 (15%) pulmonary TB, and 3 (15%) TB peritonitis. The demographic and clinical characteristics of the patients are shown in Table 1.

**Table 1:** Baseline clinical characteristics of patients with *Pneumocystis jirovecii*, candidemia, chronic disseminated candidiasis, invasive aspergillosis, mucormycosis, and TB, and of healthy volunteers.

Variable	PCP (n = 50) ^a^	Candidemia (n = 15)	CDC (n = 6) ^b^	Aspergillosis (n = 15)^c^	Mucormycosis (n = 10)^d^	TB (n = 20)^e^	Healthy volunteers (n = 20)
**Age**, years, median (IQR)	54 (43–68)	65 (52–73)	54 (36–67)	58 (50–66)	57 (48–65)	61 (46–72)	32 (30–43)
**Male gender**	31 (62)	10 (67)	4 (67)	8 (53)	4 (40)	11 (55)	8 (40)
**Underlying condition or illness**							
Diabetes	14 (28)	1 (7)	1 (17)	2 (13)	4 (40)	1 (5)	0 (0)
Solid tumor	3 (6)	10 (67)	0 (0)	3 (20)	0 (0)	0 (0)	0 (0)
Hematologic malignancy	20 (40)	0 (0)	6 (100)	8 (53)	6 (60)	3 (15)	0 (0)
Chronic renal failure	10 (20)	0 (0)	0 (0)	1 (7)	1 (10)	4 (20)	0 (0)
Rheumatologic disease	5 (10)	1 (7)	0 (0)	1 (7)	0 (0)	0 (0)	0 (0)
Transplantation	20 (40)	0 (0)	1 (17)	4 (27)	5 (50)	1 (5)	0 (0)
Liver cirrhosis	2 (4)	3 (20)	0 (0)	0 (0)	1 (10)	2 (10)	0 (0)
Neutropenia (ANC<500)	0 (0)	0 (0)	0 (0)	1 (7)	3 (30)	0 (0)	0 (0)
No underlying disease	0 (0)	0 (0)	0 (0)	1 (7)	0 (0)	2 (20)	20 (100)
Immunosuppressive condition^f^	50 (100)	15 (100)	6 (100)	13 (87)	10(100)	9 (45)	0 (0)

Abbreviations: PCP, *Pneumocystis* pneumonia; CDC, chronic disseminated candidiasis; TB, tuberculosis; IQR, interquartile range; ANC, absolute neutrophil count

Data are no. (%) patients unless otherwise indicated.

^a^Includes 47 non-HIV patients and 3 HIV patients.

^b^Includes 2 proven and 4 probable CDC

^c^Includes 6 proven and 9 probable aspergillosis.

^d^Includes 8 proven and 2 probable mucormycosis.

^e^Includes 3 pulmonary TB, 9 disseminated TB, 5 TB lymphadenopathy and 3 TB peritonitis.

^**f**^Immunosuppressive condition is defined as underlying disease such as malignancy, liver cirrhosis and chronic renal failure, and/or patients who were receiving immunosuppressive treatment.

### Diagnostic performance of the (1–3)-β-D-glucan assay

**Fig 1:**
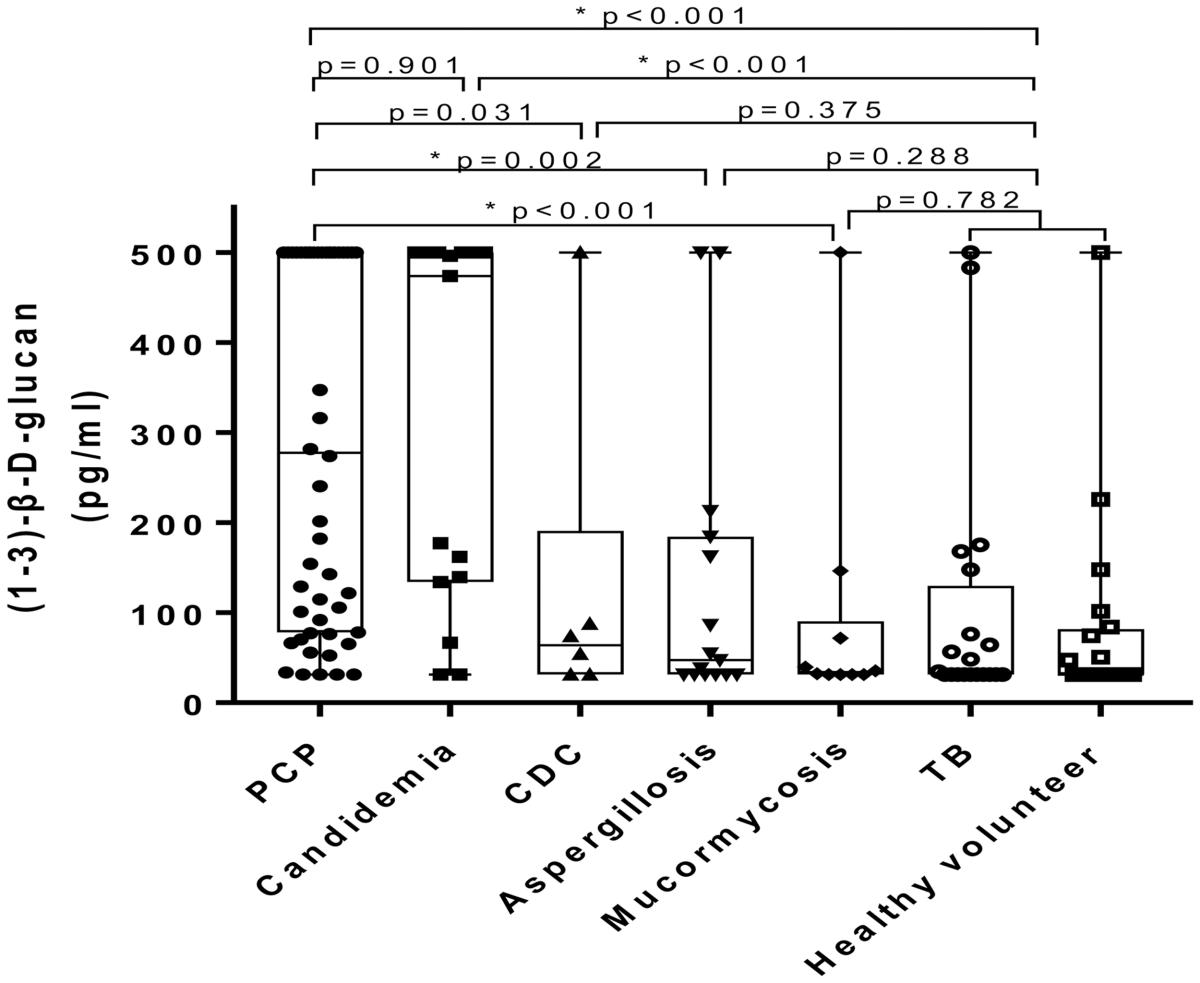
Results of Goldstream Fungus (1–3)-β-D-glucan test in the blood samples of patients with a *Pneumocystis jirovecii* infection, candidemia, CDC, invasive aspergillosis, mucormycosis, and TB, as well as healthy volunteers. Abbreviations: PCP, *Pneumocystis* pneumonia; CDC, Chronic disseminated candidiasis; TB, Tuberculosis; * *p* < 0.005.

The mean ± standard deviation values of the concentration of 1–3-β-D-glucan in the blood samples of patients with PCP (290.08 pg/mL ± 199.98) were similar to those of patients with candidemia (314.14 pg/mL ± 205.60, *p* = 0.90 at an α = 0.005) and CDC (129.74 pg/mL ± 182.79, *p* = 0.03 at an α = 0.005), but higher than those of patients with invasive aspergillosis (131.62 pg/mL ± 161.67, *p* = 0.002 at an α = 0.005), mucormycosis (95.08 pg/mL ± 146.80, *p* < 0.001 at an α = 0.005), and tuberculosis (103.31 pg/mL ± 140.81, *p* < 0.001 at an α = 0.005) as well as healthy volunteers (101.18 pg/mL ± 197.52, *p* < 0.001 at an α = 0.005). (Fig 1). Positive assay results in *P*. *jirovecii*, candidemia, CDC, invasive aspergillosis, mucormycosis, TB, and the healthy volunteers according to various cut-off values for the assay are shown in Table 2. Individual data is also listed in S1 File.

**Table 2:** Positive Goldstream Fungus (1–3)-β-D-glucan assays in patients with a *P*. *jirovecii* infection, invasive aspergillosis, mucormycosis, and TB, as well as healthy volunteers.

	PCP (n = 50)	Candidemia (n = 15)	CDC (n = 6)	Aspergillosis (n = 15)	Mucormycosis (n = 10)	TB (n = 20)	Healthy controls (n = 20)
**BG>31.25**^a^	46 (92%,95% CI 82–98)	13 (87%,95% CI 60–98)	4 (67%,95% CI 22–96)	9 (60%,95% CI32-84)	6 (60%,95% CI 26–88)	10 (50%,95% CI 27–73)	8 (40%,95% CI 19–64)
**BG>60**^b^	43 (86%,95% CI 73–94)	13 (87%,95% CI 60–98)	3 (50%,95% CI 12–88)	6 (40%,95% CI 16–68)	3 (30%,95% CI 7–65)	7 (35%,95% CI 15–59)	6 (30%,95% CI 12–54)
**BG>80**^c^	37 (74%,95% CI 60–85)	12 (80%,95% CI 52–96)	2 (33%,95% CI 4–78)	6 (40%,95% CI 16–68)	2 (20%,95% CI 3–56)	5 (25%,95% CI 9–49)	5 (25%, 95% CI 9–49)
**BG>64.21**^d^	43 (86%,95% CI 73–94)	13 (87%,95% CI 60–98)	3 (50%,95% CI 12–88)	6 (40%,95% CI 16–68)	3 (30%,95% CI 7–65)	6 (30%,95% CI 12–54)	6 (30%,95% CI 12–54)

Abbreviations: PCP, *Pneumocystis* pneumonia; CDC, chronic disseminated candidiasis; TB, TB; CI, confidence interval; BG, (1–3)-β-D-glucan.

Data are no. (%) patients unless otherwise indicated.

^a^ Optimal cut-off value with high sensitivity at the expense of specificity for PCP versus TB plus healthy control

^b^ Manufacturer-recommended cut-off point for the negative value of (1–3)-β-D-glucan assay

^c^ Manufacturer-recommended cut-off point for the positive value of (1–3)-β-D-glucan assay

^d^ Optimal cut-off value as the point of the ROC curve farthest from the diagonal line for PCP versus TB plus healthy control

Table 3 and S1–S4 Tables show the diagnostic performance of the BG assay in distinguishing patients with PCP and IFIs from the controls (healthy volunteers and patients with TB) according to various cut-off values. At a cut-off value of >80pg/mL, as recommended by the manufacturer, the assay had sensitivity of 74% (95% CI 37%-50%) and specificity of 75% (95% CI 59%-87%) for diagnosing PCP. At a cut-off value of > 31.25 pg/mL, which favors sensitivity at the expense of specificity for PCP versus TB plus healthy volunteers, the assay had sensitivity of 92% (95% CI 81%-98%) and specificity of 55% (95% CI 39%-71%) for diagnosing PCP.

**Table 3:** Diagnostic performance of the Goldstream Fungus (1–3)-β-D-glucan test in *Pneumocystis jirovecii* vs. TB plus healthy volunteers.

	Sensitivity % (n/N,^a^ 95% CI)	Specificity % (n/N,^b^ 95% CI)	PPV (95% CI)	NPV (95% CI)	Positive likelihood ratio (95% CI)	Negative likelihood ratio (95% CI)
**BG>31.25**^c^	92 (46/50, 81–98)	55 (22/40, 38–71)	72 (59–82)	85 (65–96)	2.04 (1.44–2.91)	6.88 (2.58–18.33)
**BG>60**^d^	86 (43/50, 73–94)	68 (27/40, 51–81)	77 (64–87)	79 (62–91)	2.65 (1.67–4.19)	4.82 (2.35–9.90)
**BG>64.21**^e^	84 (42/50, 71–93)	70 (28/40, 53–83)	78 (64–88)	78 (61–90)	2.8 (1.72–4.56)	4.38 (2.25–8.52)
**BG>80**^f^	74 (37/50, 60–85)	75 (30/40, 59–87)	79 (64–89)	70 (54–83)	2.96 (1.69–5.19)	2.88 (1.75–4.76)

Abbreviations: PCP, *Pneumocystis* pneumonia; TB, TB, CI, confidence interval; BG, (1–3)-β-D-glucan.

Data are no. (%) patients unless otherwise indicated.

^a^ Sensitivity was determined by dividing the no of patients with a positive test results by the number of patients with PCP tested

^b^ Specificity was determined by dividing the no of patients with a negative test results by the number of healthy control tested

^c^ Optimal cut-off value with high sensitivity at the expense of specificity for PCP versus TB plus healthy control

^d^ Manufacturer-recommended cut-off point for the negative value of the (1–3)-β-D-glucan

^e^ Optimal cut-off value as the point of the ROC curve farthest from the diagonal line for PCP versus TB plus healthy control

^f^ Manufacturer-recommended cut-off point for the positive value of the (1–3)-β-D-glucan

## Discussion

The diagnosis of PCP relies on microscopic visualization of *P*. *jirovecii* or detection of its DNA in respiratory specimens, which needs invasive procedures such as bronchoalveolar lavage. We therefore measured the diagnostic utility of blood levels of BG as a non-invasive screening test in patients with PCP. At a cut-off value of >64.21 pg/mL, which was farthest from the diagonal line of the ROC curve for PCP versus controls consisting of tuberculosis patients and healthy volunteers, the BG assay had a sensitivity of 87%, a specificity of 70%, PPV of 78% and NPV of 78% (Table 3). Recently, a meta-analysis evaluating 11 retrospective studies of patients with laboratory-confirmed PCP and at-risk patient controls found pooled sensitivities and specificities of 95% and 86%, respectively, for detecting BG in cases of proven PCP [17]. Therefore, our findings are consistent with previous data and indicate that the BG assay could be useful for ruling out PCP.

However, the specificity of the BG assay in our study was lower than that of the BG test results in the meta-analysis data. The reason for this discrepancy is not clear. Possible explanations might include differences in study design (i.e., the exclusion of invasive fungal infections), the target population, and the type of BG assay used. In addition, various factors such as hemodialysis through cellulose membranes, the use of certain types of gauzes, certain blood products, gram-negative endotoxemia, antibiotics, and severe mucositis have been associated with false-positive results on BG assays [18]. Therefore, a positive BG result in critically ill patients who have these factors must be interpreted with caution. In contrast, the conventional assays for the diagnosis of PCP, which are performed using BAL samples, have an excellent sensitivity and specificity. The sensitivity and specificity of immunofluorescence staining with monoclonal antibodies are both 100% [19], while those of polymerase chain reaction are 100% and 94%, respectively [19]. Therefore, conventional assays in BAL fluid may be the preferred tests for the confirmation of PCP in patients with suspected PCP, even when positive BG results are reported.

If the diagnosis of PCP is missed, PCP is fatal in the absence of trimethoprim-sulfamethoxazole treatment. Therefore, the penalty of missing the diagnosis is so high that a diagnostic test with a high sensitivity is needed. Invasive bronchoscopic examinations are required to obtain BAL fluid for the confirmation of PCP, and it would be useful for clinicians to have access to a non-invasive confirmatory test with a high specificity to reduce false-positive results. In this context, the determination of a cut-off value offering an optimal balance between sensitivity and specificity is needed in patients with suspected PCP. Therefore, a lower cut-off value may need to be cautiously applied to improve the sensitivity of BG assays in various clinical scenarios.

BG is a cell wall component of various fungal organisms such as candida and aspergillus; hence the BG assay has been proposed as a screening test for these fungi. We measured BG levels in several invasive fungal diseases. As expected, the mean ± standard deviation values of the concentration of BG in the blood samples of patients with candidemia (314.14 pg/mL ± 205.60) were elevated similarly to those of patients with PCP (290.08 pg/mL ± 199.98, *p* = 0.90). It is also worth mentioning the results of the BG assay in patients with CDC, which is classified as a deep-seated candidiasis. The positive rate of blood culture in CDC is <20% [20]. In a recent prospective evaluation of serum BG assays in patients with probable or proven fungal disease, 5 CDC patients without concomitant candidemia all had positive BG results at the time of diagnosis [21]. Furthermore, in the 3 patients with CDC for whom follow-up BG results were available, the BG values remained high for more than 6 weeks [21]. This implies that patients with slowly decreasing BG profiles are more likely to have deep-seated infections [21]. In contrast, we found that only two of six patients with CDC showed a positive result on BG assay (BG > 80 pg/mL) in the present study.

It is worth noting that the BG assay may not detect BG in subjects with mucormycosis, because the agents of mucormycosis theoretically do not produce BG. However, there are limited data on whether BG can be detected in these patients in real clinical practice. Until now, only one of four patients with mucormycosis present in three study cohorts gave positive results in the BG assay [9, 12, 22]. We evaluated 10 patients with mucormycosis including 8 proven and 2 probable cases. Of these 10 patients, two showed positive results on BG assay (BG > 80 pg/mL). The reason for this observation is not clear. Possible explanations are co-infections or false positive due to causes such as intravenous antibiotics [21]. In addition, decreasing the cut-off value of the BG concentration comes at the cost of an increased frequency of false-positive results. Given that there is no known optimal cut-off value for negative BG test results in patients with mucormycosis, it is possible that positive BG assay results depending on various cut-off values might simply represent false-positive results associated with lowering cut-off values. Further studies are needed in this area.

This study has some limitations. First, we used data on healthy individuals and tuberculosis patients as controls. Because these individuals are not representative of the populations to which the test would be applied in clinical practice, the prevalences of diseases measured in our study may not reflect the actual prevalences in real clinical practice. Therefore, caution is needed when interpreting our positive predictive values and negative predictive values. Second, of the 50 patients with *P*. *jirovecii*, 3 were HIV positive and 47 were HIV-negative. Since most of the patients with PCP were HIV negative, it is difficult to generalize our findings to PCP patients with HIV. Nakamura and colleagues have suggested that the detection rate of BG in HIV negative patients is lower than in HIV positive patients [23], while Onishi and colleagues have demonstrated that diagnosis with the BG assay is useful regardless of HIV status [24]. Further meta-analysis without high interstudy heterogeneity would help to clarify this issue. Third, some may argue that missed diagnoses of PCP or *P*. *jirovecii* colonization may affect the results of our study. The absence of PCP in immunocompromised patients cannot be confirmed without performing invasive tests. Therefore, false-positive results from the BG assay may result from the missed diagnosis of PCP. However, among the 46 patients who were classified into the non-PCP group in our study, 10 patients underwent bronchoscopy and PCP IHC or PCR results from BAL fluid were available for 5 patients. All of those 5 patients showed negative results from PCP IHC or PCR tests of their BAL fluid. In addition, *P*. *jirovecii* colonization is possible in healthy persons as well as in immunocompromised patients [25]. Therefore, false-positive results obtained from the BG assay in healthy volunteers as well as in patients with mucormycosis or TB may result from *P*. *jirovecii* colonization. However, a previous study demonstrated that the serum BG concentration was significantly higher in patients with PCP than in patients with *P*. *jirovecii* colonization [26]. Further studies are needed on this subject. Lastly, although there are many similarities between the various BG assays such as Fungitell, Fungitec and the Goldstream Fungus (1–3)-β-D-glucan test, differences in diagnostic performance may exist [17].

In conclusion, the BG assay appears to be a rapid and noninvasive adjunct test for diagnosing PCP. The assay could also be a useful approach for diagnosing deep-seated fungal infections such as CDC.

## Supporting information

S1 Table Diagnostic performance of the Goldstream Fungus (1–3)-β-D-glucan test in candidemia vs TB plus healthy volunteer. Abbreviations: PCP, *Pneumocystis* pneumonia; TB, TB, CI, confidence interval; BG, (1–3)-β-D-glucan. Data are no. (%) patients unless otherwise indicated. ^a^ Sensitivity was determined by dividing the no of patients with a positive test results by the number of patients with PCP tested. ^b^ Specificity was determined by dividing the no of patients with a negative test results by the number of healthy control tested. ^c^ Optimal cut-off value with high sensitivity at the expense of specificity for candidemia versus TB plus healthy control. ^d^ Manufacturer-recommended cut-off point for the negative value of the (1–3)-β-D-glucan. ^e^ Manufacturer-recommended cut-off point for the positive value of the (1–3)-β-D-glucan. ^f^ Optimal cut-off value as the point of the ROC curve farthest from the diagonal line for candidemia versus TB plus healthy control.
(DOCX)

S2 Table Diagnostic performance of the Goldstream Fungus (1–3)-β-D-glucan test in CDC vs TB plus healthy volunteer. Abbreviations: CDC, chronic disseminated candidiasis; TB, TB; CI, confidence interval; BG, (1–3)-β-D-glucan. Data are no. (%) patients unless otherwise indicated. ^a^ Sensitivity was determined by dividing the no of patients with a positive test results by the number of patients with PCP tested. ^b^ Specificity was determined by dividing the no of patients with a negative test results by the number of healthy control tested. ^c^ Optimal cut-off value with high sensitivity at the expense of specificity for PCP versus TB plus healthy control. ^d^ Manufacturer-recommended cut-off point for the negative value of the (1–3)-β-D-glucan. ^e^ Manufacturer-recommended cut-off point for the positive value of the (1–3)-β-D-glucan. ^f^ Optimal cut-off value as the point of the ROC curve farthest from the diagonal line for CDC versus TB plus healthy control.
(DOCX)

S3 Table Diagnostic performance of the Goldstream Fungus (1–3)-β-D-glucan test in aspergillosis vs TB plus healthy volunteer. Abbreviations: PCP, *Pneumocystis* pneumonia; TB, TB, CI, confidence interval; BG, (1–3)-β-D-glucan. Data are no. (%) patients unless otherwise indicated. ^a^ Sensitivity was determined by dividing the no of patients with a positive test results by the number of patients with PCP tested. ^b^ Specificity was determined by dividing the no of patients with a negative test results by the number of healthy control tested. ^c^ Optimal cut-off value with high sensitivity at the expense of specificity for PCP versus TB plus healthy control. ^d^ Manufacturer-recommended cut-off point for the negative value of the (1–3)-β-D-glucan. ^e^ Manufacturer-recommended cut-off point for the positive value of the (1–3)-β-D-glucan. ^f^ Optimal cut-off value as the point of the ROC curve farthest from the diagonal line for aspergillosis versus TB plus healthy control.
(DOCX)

S4 Table Diagnostic performance of the Goldstream Fungus (1–3)-β-D-glucan test in mucormycosis vs TB plus healthy volunteer. Abbreviations: PCP, *Pneumocystis* pneumonia; TB, TB, CI, confidence interval; BG, (1–3)-β-D-glucan. Data are no. (%) patients unless otherwise indicated. ^a^ Sensitivity was determined by dividing the no of patients with a positive test results by the number of patients with PCP tested. ^b^ Specificity was determined by dividing the no of patients with a negative test results by the number of healthy control tested. ^c^ Optimal cut-off value with high sensitivity at the expense of specificity for PCP versus TB plus healthy control. ^d^ Manufacturer-recommended cut-off point for the negative value of the (1–3)-β-D-glucan. ^e^ Manufacturer-recommended cut-off point for the positive value of the (1–3)-β-D-glucan. ^f^ Optimal cut-off value as the point of the ROC curve farthest from the diagonal line for mucormycosis versus TB plus healthy control.
(DOCX)

S1 File Individual values of the (1–3)-β-D-glucan assay in patients with Pneumocystis jirovecii compared with those with candidiasis, aspergillosis, mucormycosis, and tuberculosis, and healthy volunteers. (XLSX)
