# Supplementary material for: Diagnostic performance of the (1–3)-β-D-glucan assay in patients with Pneumocystis jirovecii compared with those with candidiasis, aspergillosis, mucormycosis, and tuberculosis, and healthy volunteers
Source: PLoS One. 2017 Nov 30;12(11):e0188860. doi: 10.1371/journal.pone.0188860 (PMC5708637; doi:10.1371/journal.pone.0188860)
Supplement: S1 Table — Abbreviations: PCP, Pneumocystis pneumonia; TB, TB, CI, confidence interval; BG, (1–3)-β-D-glucan. Data are no. (%) patients unless otherwise indicated. a Sensitivity was determined by dividing the no of patients with a positive test results by the number of patients with PCP tested. b Specificity was determined by dividing the no of patients with a negative test results by the number of healthy control tested. c Optimal cut-off value with high sensitivity at the expense of specificity for candidemia versus TB plus healthy control. d Manufacturer-recommended cut-off point for the negative value of the (1–3)-β-D-glucan. e Manufacturer-recommended cut-off point for the positive value of the (1–3)-β-D-glucan. f Optimal cut-off value as the point of the ROC curve farthest from the diagonal line for candidemia versus TB plus healthy control. (DOCX) [file pone.0188860.s001.docx]

**S1 Table.** **Diagnostic performance of the Goldstream Fungus (1–3)-β-D-glucan test in Candidemia vs TB plus healthy volunteer**

|  | **Sensitivity %**  **(n/N,^a^ 95% CI)** | **Specificity %**  **(n/N,^b^ 95% CI)** | **PPV**  **(95% CI)** | **NPV**  **(95% CI)** | **Positive likelihood**  **ratio (95% CI)** | **Negative likelihood**  **ratio (95% CI)** |
| --- | --- | --- | --- | --- | --- | --- |
| **BG>31.25^c^** | 87  (13/15, 60-98) | 55  (22/40, 38-71) | 42  (25-61) | 92  (73-99) | 1.93  (1.30-2.86) | 4.13  (1.10-15.45) |
| **BG>60^d^** | 87  (13/15, 60-98) | 68  (27/40, 51-81) | 50  (30-70) | 93  (77-99) | 2.67  (1.64-4.35) | 5.06  (1.37-18.73) |
| **BG>80^e^** | 80  (12/15, 52-96) | 75  (30/40, 59-87) | 55  (32-76) | 91  (76-98) | 3.20  (1.77-5.79) | 3.75  (1.34-10.48) |
| **BG>101.33^f^** | 80  (12/15, 52-96) | 80  (32/40, 64-91) | 60  (36-81) | 91  (77-98) | 4.00  (2.05-7.81) | 4.00  (1.44-11.14) |

Abbreviations: PCP, *Pneumocystis* pneumonia; TB, TB, CI, confidence interval; BG, (1–3)-β-D-glucan.

Data are no. (%) patients unless otherwise indicated.

^a^ Sensitivity was determined by dividing the no of patients with a positive test results by the number of patients with PCP tested

^b^ Specificity was determined by dividing the no of patients with a negative test results by the number of healthy control tested

^c^ Optimal cut-off value with high sensitivity at the expense of specificity for candidemia versus TB plus healthy control

^d^ Manufacturer-recommended cut-off point for the negative value of the (1–3)-β-D-glucan

^e^ Manufacturer-recommended cut-off point for the positive value of the (1–3)-β-D-glucan

^f^ Optimal cut-off value as the point of the ROC curve farthest from the diagonal line for candidemia versus TB plus healthy control
