# Supplementary material for: Diagnostic performance of the (1–3)-β-D-glucan assay in patients with Pneumocystis jirovecii compared with those with candidiasis, aspergillosis, mucormycosis, and tuberculosis, and healthy volunteers
Source: PLoS One. 2017 Nov 30;12(11):e0188860. doi: 10.1371/journal.pone.0188860 (PMC5708637; doi:10.1371/journal.pone.0188860)
Supplement: S2 Table — Abbreviations: CDC, chronic disseminated candidiasis; TB, TB; CI, confidence interval; BG, (1–3)-β-D-glucan. Data are no. (%) patients unless otherwise indicated. a Sensitivity was determined by dividing the no of patients with a positive test results by the number of patients with PCP tested. b Specificity was determined by dividing the no of patients with a negative test results by the number of healthy control tested. c Optimal cut-off value with high sensitivity at the expense of specificity for PCP versus TB plus healthy control. d Manufacturer-recommended cut-off point for the negative value of the (1–3)-β-D-glucan. e Manufacturer-recommended cut-off point for the positive value of the (1–3)-β-D-glucan. f Optimal cut-off value as the point of the ROC curve farthest from the diagonal line for CDC versus TB plus healthy control. (DOCX) [file pone.0188860.s002.docx]

**S2 Table. Diagnostic performance of the Goldstream Fungus (1–3)-β-D-glucan test in CDC vs TB plus healthy volunteer**

|  | **Sensitivity %**  **(n/N,^a^ 95% CI)** | **Specificity %**  **(n/N,^b^ 95% CI)** | **PPV**  **(95% CI)** | **NPV**  **(95% CI)** | **Positive likelihood**  **ratio (95% CI)** | **Negative likelihood**  **ratio (95% CI)** |
| --- | --- | --- | --- | --- | --- | --- |
| **BG>31.25^c^** | 67  (4/6, 22-96) | 55  (22/40, 38-71) | 18  (5-40) | 92  (73-99) | 1.48  (0.76-2.87) | 1.65  (0.51-5.29) |
| **BG>60^d^** | 50  (3/6, 12-88) | 68  (27/40, 51-81) | 19  (4-46) | 90  (73-98) | 1.54  (0.62-3.85) | 1.35  (0.59-3.09) |
| **BG>80^e^** | 33  (2/6, 4-78) | 75  (30/40, 59-87) | 17  (2-48) | 88  (73-97) | 1.33  (0.37-4.67) | 1.13  (0.62-2.03) |
| **BG>50.79^f^** | 67  (4/6, 22-96) | 65  (26/40, 48-79) | 22  (6-48) | 93  (77-99) | 1.90  (0.94-3.86) | 1.95  (0.61-6.18) |

Abbreviations: CDC, chronic disseminated candidiasis; TB, TB; CI, confidence interval; BG, (1–3)-β-D-glucan.

Data are no. (%) patients unless otherwise indicated.

^a^ Sensitivity was determined by dividing the no of patients with a positive test results by the number of patients with PCP tested

^b^ Specificity was determined by dividing the no of patients with a negative test results by the number of healthy control tested

^c^ Optimal cut-off value with high sensitivity at the expense of specificity for PCP versus TB plus healthy control

^d^ Manufacturer-recommended cut-off point for the negative value of the (1–3)-β-D-glucan

^e^ Manufacturer-recommended cut-off point for the positive value of the (1–3)-β-D-glucan

^f^ Optimal cut-off value as the point of the ROC curve farthest from the diagonal line for CDC versus TB plus healthy control
