# Supplementary material for: Diagnostic performance of the (1–3)-β-D-glucan assay in patients with Pneumocystis jirovecii compared with those with candidiasis, aspergillosis, mucormycosis, and tuberculosis, and healthy volunteers
Source: PLoS One. 2017 Nov 30;12(11):e0188860. doi: 10.1371/journal.pone.0188860 (PMC5708637; doi:10.1371/journal.pone.0188860)
Supplement: S3 Table — Abbreviations: PCP, Pneumocystis pneumonia; TB, TB, CI, confidence interval; BG, (1–3)-β-D-glucan. Data are no. (%) patients unless otherwise indicated. a Sensitivity was determined by dividing the no of patients with a positive test results by the number of patients with PCP tested. b Specificity was determined by dividing the no of patients with a negative test results by the number of healthy control tested. c Optimal cut-off value with high sensitivity at the expense of specificity for PCP versus TB plus healthy control. d Manufacturer-recommended cut-off point for the negative value of the (1–3)-β-D-glucan. e Manufacturer-recommended cut-off point for the positive value of the (1–3)-β-D-glucan. f Optimal cut-off value as the point of the ROC curve farthest from the diagonal line for aspergillosis versus TB plus healthy control. (DOCX) [file pone.0188860.s003.docx]

**S3 Table. Diagnostic performance of the** **Goldstream Fungus (1–3)-β-D-glucan test in Aspergillosis vs TB plus healthy volunteer**

|  | **Sensitivity %**  **(n/N,^a^ 95% CI)** | **Specificity %**  **(n/N,^b^ 95% CI)** | **PPV**  **(95% CI)** | **NPV**  **(95% CI)** | **Positive likelihood**  **ratio (95% CI)** | **Negative likelihood**  **ratio (95% CI)** |
| --- | --- | --- | --- | --- | --- | --- |
| **BG>31.25^c^** | 60  (9/15, 32-84) | 55  (22/40, 38-71) | 33  (17-54) | 79  (59-92) | 1.33  (0.78-2.28) | 1.38  (0.70-2.71) |
| **BG>60^d^** | 40  (6/15, 16-68) | 68  (27/40, 51-81) | 32  (13-57) | 75  (58-88) | 1.23  (0.57-2.64) | 1.23  (0.71-1.79) |
| **BG>80^e^** | 40  (6/15, 16-68) | 75  (30/40, 59-87) | 38  (15-65) | 77  (61-89) | 1.60  (0.70-3.63) | 1.25  (0.80-1.96) |
| **BG>147.92^f^** | 87  (5/15 , 60-98) | 85  (34/40, 70-94) | 45  (17-77) | 77  (62-89) | 2.22  (0.80-6.21) | 1.28  (0.87-1.87) |

Abbreviations: PCP, *Pneumocystis* pneumonia; TB, TB, CI, confidence interval; BG, (1–3)-β-D-glucan.

Data are no. (%) patients unless otherwise indicated.

^a^ Sensitivity was determined by dividing the no of patients with a positive test results by the number of patients with PCP tested

^b^ Specificity was determined by dividing the no of patients with a negative test results by the number of healthy control tested

^c^ Optimal cut-off value with high sensitivity at the expense of specificity for PCP versus TB plus healthy control

^d^ Manufacturer-recommended cut-off point for the negative value of the (1–3)-β-D-glucan

^e^ Manufacturer-recommended cut-off point for the positive value of the (1–3)-β-D-glucan

^f^ Optimal cut-off value as the point of the ROC curve farthest from the diagonal line for aspergillosis versus TB plus healthy control
