# Supplementary material for: Diagnostic performance of the (1–3)-β-D-glucan assay in patients with Pneumocystis jirovecii compared with those with candidiasis, aspergillosis, mucormycosis, and tuberculosis, and healthy volunteers
Source: PLoS One. 2017 Nov 30;12(11):e0188860. doi: 10.1371/journal.pone.0188860 (PMC5708637; doi:10.1371/journal.pone.0188860)
Supplement: S4 Table — Abbreviations: PCP, Pneumocystis pneumonia; TB, TB, CI, confidence interval; BG, (1–3)-β-D-glucan. Data are no. (%) patients unless otherwise indicated. a Sensitivity was determined by dividing the no of patients with a positive test results by the number of patients with PCP tested. b Specificity was determined by dividing the no of patients with a negative test results by the number of healthy control tested. c Optimal cut-off value with high sensitivity at the expense of specificity for PCP versus TB plus healthy control. d Manufacturer-recommended cut-off point for the negative value of the (1–3)-β-D-glucan. e Manufacturer-recommended cut-off point for the positive value of the (1–3)-β-D-glucan. f Optimal cut-off value as the point of the ROC curve farthest from the diagonal line for mucormycosis versus TB plus healthy control. (DOCX) [file pone.0188860.s004.docx]

**S4 Table. Diagnostic performance of the Goldstream Fungus (1–3)-β-D-glucan test in Mucormycosis vs TB plus healthy volunteer**

|  | **Sensitivity %**  **(n/N,^a^ 95% CI)** | **Specificity %**  **(n/N,^b^ 95% CI)** | **PPV**  **(95% CI)** | **NPV**  **(95% CI)** | **Positive likelihood**  **ratio (95% CI)** | **Negative likelihood**  **ratio (95% CI)** |
| --- | --- | --- | --- | --- | --- | --- |
| **BG>31.25^c, f^** | 60  (6/10, 26-88) | 55  (22/40, 38-71) | 25  (10-47) | 85  (65-96) | 1.33  (0.72-2.46) | 1.38  (0.61-3.09) |
| **BG>60^d^** | 30  (3/10, 7-65) | 68  (27/40, 51-81) | 19  (4-46) | 79  (62-91) | 0.92  (0.32-2.63) | 0.96  (0.61-1.53) |
| **BG>80^e^** | 20  (2/10, 3-56) | 75  (30/40, 59-87) | 17  (2-48) | 79  (63-90) | 0.80  (0.21-3.09) | 0.93  (0.66-1.34) |

Abbreviations: PCP, *Pneumocystis* pneumonia; TB, TB, CI, confidence interval; BG, (1–3)-β-D-glucan.

Data are no. (%) patients unless otherwise indicated.

^a^ Sensitivity was determined by dividing the no of patients with a positive test results by the number of patients with PCP tested

^b^ Specificity was determined by dividing the no of patients with a negative test results by the number of healthy control tested

^c^ Optimal cut-off value with high sensitivity at the expense of specificity for PCP versus TB plus healthy control

^d^ Manufacturer-recommended cut-off point for the negative value of the (1–3)-β-D-glucan

^e^ Manufacturer-recommended cut-off point for the positive value of the (1–3)-β-D-glucan

^f^ Optimal cut-off value as the point of the ROC curve farthest from the diagonal line for mucormycosis versus TB plus healthy control
